# A Major Facilitator Superfamily Transporter Contributes to Ergot Alkaloid Accumulation but Not Secretion in *Aspergillus leporis*

**DOI:** 10.3390/applmicrobiol4010028

**Published:** 2024-02-20

**Authors:** Abigail M. Jones, Kyle A. Davis, Daniel G. Panaccione

**Affiliations:** Division of Plant and Soil Sciences, West Virginia University, Morgantown, WV 26506, USA;

**Keywords:** ergot alkaloids, major facilitator superfamily, transporter, lysergic acid, lysergic acid amides, *Aspergillus*

## Abstract

Ergot alkaloids are fungal natural products with important roles in agriculture and medicine. We used heterologous expression and gene knockout approaches to investigate potential roles for the product of a major facilitator superfamily transporter gene (*easT*) recently found in an ergot alkaloid biosynthetic gene cluster in *Aspergillus leporis*. A strain of *Aspergillus fumigatus* previously engineered to accumulate lysergic acid, but which did not convert the precursor agroclavine to lysergic acid efficiently or secrete lysergic acid well, was chosen as an expression host for *easT*. Expression of *easT* in this strain resulted in accumulation of significantly more pathway intermediates but no detectable lysergic acid. Secretion of ergot alkaloids was reduced in the *easT*-expressing strain. EasT localized to discrete vesicle-like structures in the cytosol of *A. fumigatus*, with no localization detected in the plasma membrane. When *easT* was knocked out in *A. leporis*, accumulation of lysergic acid amides was reduced relative to the wild type. There was no negative effect on secretion of ergot alkaloids in the knockout mutant. The data indicate that *easT* encodes a product that contributes to accumulation of ergot alkaloids, perhaps by transporting intermediates between cellular compartments, but does not have a significant role in secreting ergot alkaloids.

## 1. Introduction

Ergot alkaloids are specialized metabolites produced by a range of fungi. These compounds are important in agriculture, where they contaminate grains and forage crops, and in medicine, where they serve as the foundations of pharmaceuticals used to treat migraines, dementia, hyperprolactinemia, and other conditions [1–3]. The roles of various genes in different branches of the ergot alkaloid pathway have been studied extensively [3–5]. Knowledge of the biosynthetic pathways may be applied to control accumulation of the compounds in agriculture or exploited to produce pharmaceuticals or their lead compounds more efficiently.

Some ergot alkaloid-producing fungi (e.g., *Metarhizium brunneum*, *Aspergillus homomorphus*, and *Aspergillus leporis*) secrete most of their ergot alkaloids [5,6], whereas other species (most notably the opportunistic human pathogen *Aspergillus fumigatus*) retain the majority of their ergot alkaloids in the solid phase of the producing fungus [7,8]. We know very little about how secretion of ergot alkaloids is determined or the role of any particular transporter in the biosynthesis or secretion of these important compounds. Previous studies suggest secretion is a function of the producing fungus rather than a function of the alkaloid. For example, dihydrolysergic acid is an ergot alkaloid not naturally found in either *A. fumigatus* or *M. brunneum*. Strains of *A. fumigatus* engineered to produce dihydrolysergic acid retained the vast majority of it in the solid phase of the culture, whereas a strain of *M. brunneum* engineered to produce dihydrolysergic acid secreted over 80% of the alkaloid into its growth medium [8]. Similarly, lysergic acid, an ergot alkaloid that is foreign to *A. fumigatus* and a transient intermediate in *M. brunneum*, is also retained in colonies of *A. fumigatus* engineered to produce this compound but secreted at a rate over 90% in a lysergic acid over-producing mutant of *M. brunneum* [8].

**Figure 1.**
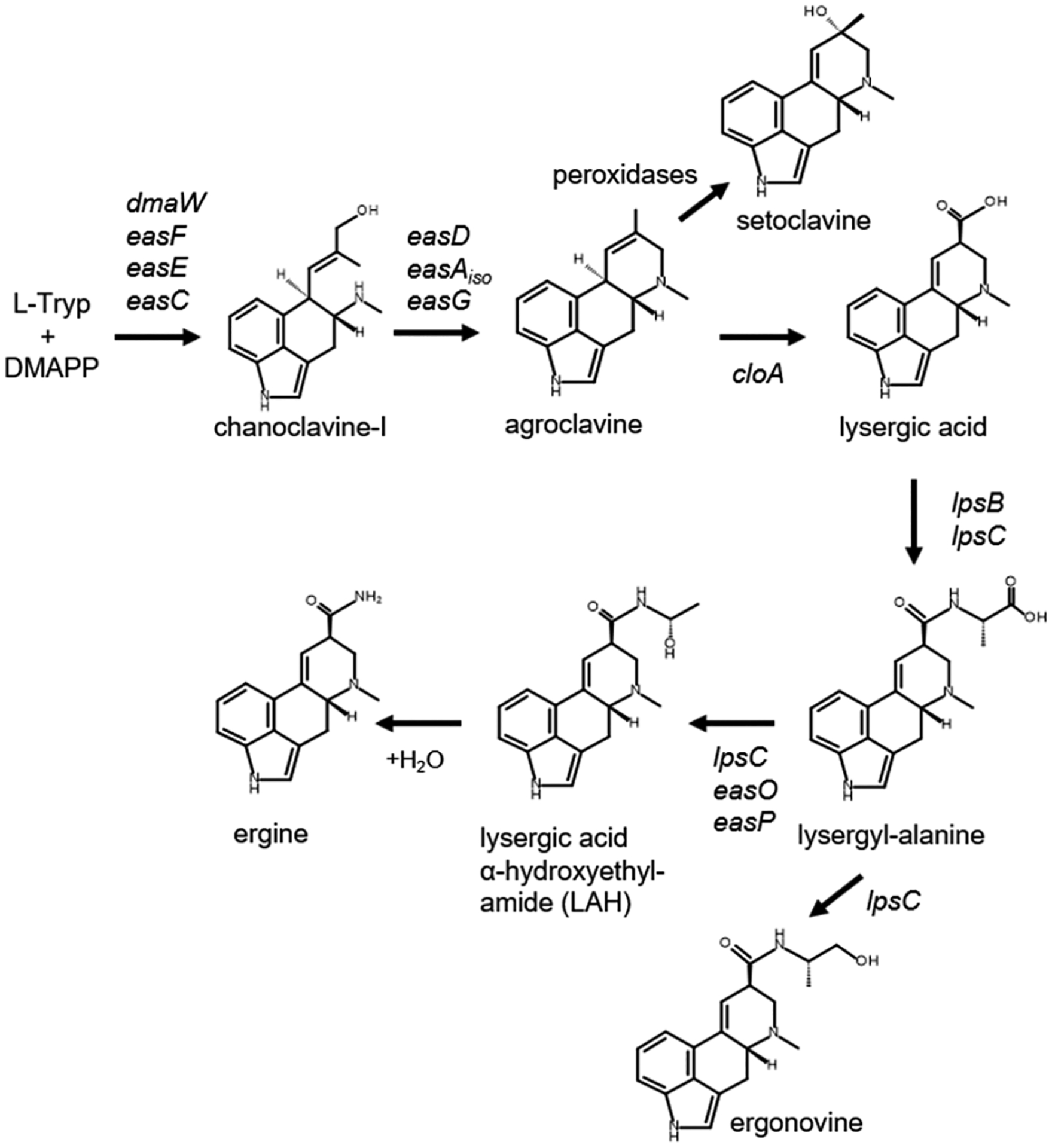
Pathway to lysergic acid amides. Genes controlling relevant steps are indicated. Setoclavine is derived by oxidation of accumulated agroclavine by peroxidases common to many microorganisms [19–21]. Abbreviations: Tryp, tryptophan; DMAPP, dimethylallylpyrophosphate.

The literature contains few studies on the subcellular location of some of the steps in ergot alkaloid synthesis in fungi. While expressing genes from *Aspergillus japonicus* in *Saccharomyces cerevisiae*, Nielsen et al. [9] noted a requirement for the N-terminal signal sequence of the enzyme EasE for synthesis of chanoclavine-I, suggesting localization to, or oxidative folding of, the protein in the endoplasmic reticulum (ER). These same authors observed the dispensability of a putative C-terminal peroxisomal-localizing sequence on the enzyme EasC [9]. Also working with *S. cerevisiae* as a heterologous expression host, Wu et al. [10] presented evidence that enzymes expressed from the first seven genes of the ergot alkaloid pathway (resulting in production of agroclavine; Figure 1) were found in different locations in *S. cerevisiae*. The sources of these enzymes included *Aspergillus japonicus* (DmaW, EasE, and EasC), *Aspergillus fumigatus* (EasF), *Claviceps fusiformis* (EasA), and *Claviceps purpurea* (EasD and EasG). In transformed yeast, EasC and EasF were observed in the cytosol, DmaW and EasA were found in peroxisomes, EasE in the ER, and EasD and EasG in mitochondria. Sequences of the enzymes (except for DmaW) contained elements indicating that the localization observed in *S. cerevisiae* represents their localization in the source fungi [10]. The investigators suggested that shuttling of intermediates between these compartments represents a significant bottleneck in ergot alkaloid biosynthesis. In support of this hypothesis, relocalizing EasD, EasA, and EasG to the ER of an engineered yeast strain improved agroclavine accumulation significantly [10].

Research on other fungal specialized metabolite pathways demonstrates localization of certain pathway steps and the implicit need for shuttling of intermediates between compartments. Studies of the aflatoxin pathway of *Aspergillus parasiticus* have indicated localization of multiple steps in relatively large, vesicle-like structures named aflatoxisomes [11]. Similarly, steps in the synthesis of trichothecenes in *Fusarium graminearum* are localized in membrane-bound structures named toxisomes that appear to result from re-organization of the endoplasmic reticulum [12–15]. Compartmentalization of some pathway steps, and failure to appropriately compartmentalize heterologously expressed enzymes, has been offered as one possible explanation for poor turnover of certain ergot alkaloid pathway intermediates to end-products in engineered fungi, particularly when expressing genes from one pathway branch in a recipient typically having another [8,15].

*Aspergillus leporis* was originally isolated from rabbit dung [16] and has more recently been observed in the rhizospheres of selenium-tolerant plants [17] and as an opportunistic pathogen of insects [18]. This fungus produces ergot alkaloids from several branches of the ergot alkaloid pathway, including the lysergic acid derivative lysergic acid α-hydroxyethylamide (LAH) (Figure 1) [5]. Characterization of the ergot alkaloid gene clusters of *A. leporis* revealed the presence of a major facilitator superfamily transporter-encoding gene, named *easT*, in one of the LAH-associated gene clusters [6]. Interestingly, a homolog of this gene that is located in an ergot alkaloid synthesis cluster of the closely related fungus *A. hancockii* was pseudogenized, and *A. hancockii* accumulated lower concentrations of ergot alkaloids and secreted only a small portion of them compared to *A. leporis* [6]. The objective of this present study was to analyze the potential function of EasT in the synthesis and secretion of ergot alkaloids via heterologous expression and gene knockout approaches.

## 2. Materials and Methods

### 2.1. Heterologous Expression of easT in A. fumigatus Strain LA

Nucleotides 26,176 to 24,143 (in reverse orientation) of *A. leporis* scaffold 79, contig 104 (GenBank accession SWBU01000104.1), including the complete coding sequences of *easT* along 297 bp of 3′ non-translated sequences (3′-UTR), were fused to the *easA*/*easG* promoter of *A. fumigatus* Af293 (nt 2,908,140 to 2,908,132 of GenBank accession AAHF01000001.1) by fusion PCR. Primers and relevant details of the PCRs are included in Table S1. The basic PCR conditions used throughout this study were as follows. PCRs were conducted with Phusion green master mix with approximately 20 ng of fungal genomic DNA or 1 ng of gel-purified PCR product as template in a program consisting of two min at 98 °C, followed by 35 cycles of 15 s at 98 °C, 15 s at the temperature listed in Table S1 for primer annealing, and polymerization at 72 °C for the length of time indicated in Table S1. The expression host chosen for this construct was *A. fumigatus* strain LA, a derivative of *A. fumigatus* isolate FGSC A1141 that had been modified to accumulate lysergic acid as its ergot alkaloid pathway end-product [21]. Protoplasts were prepared and transformed with the *easT* construct according to methods described previously [21,22]. Transformants were purified to nuclear homogeneity by culturing from single conidia and tested for the presence of the introduced construct by PCR with primer combination 3 (Table S1).

Stationary cultures (*n* = 6 per treatment) of the recipient strain (*A. fumigatus* strain LA) and an *easT*-expressing transformant were grown in 5 mL of malt extract broth (per liter: 6.0 g malt extract, 1.8 g maltose, 6.0 g D-glucose, and 1.2 g yeast extract), inoculated with 500,000 conidia, in 60 mm Petri dishes at 37 °C for 14 days. Liquid and solid phases were separated at harvest by vacuum filtration through pre-weighed nylon membranes (0.2 μM pore size). The volume of the liquid phase was measured before diluting an aliquot with an equal volume of methanol for analysis by high-performance liquid chromatography (HPLC). The solid phase was dried on the membrane and weighed before a weighed portion of the dried fungus was removed and extracted in methanol (50 μL per mg of dried fungus). Percent secretion of a given alkaloid was calculated by dividing moles of alkaloid in the liquid phase by the sum of the moles of alkaloid in the solid and liquid phases and multiplying the quotient by 100.

Ergot alkaloids were analyzed by HPLC, as described in detail by Robinson and Panaccione [21]. Briefly, the alkaloids were separated by reverse-phase chromatography with the solid phase as an ODS3 column (5 μm particle size, 50 mm × 4.6 mm inside diameter; Phenomenex, Torrance, CA, USA) and the mobile phase as a multilinear, binary gradient from 5% acetonitrile + 95% aqueous ammonium acetate (50 mM) to 75% acetonitrile + 25% aqueous ammonium acetate (50 mM). The identities of peaks corresponding to chanoclavine-I, agroclavine, setoclavine, and lysergic acid were established previously by studies with high-resolution liquid chromatography–mass spectrometry (LC-MS) and authentic (chanoclavine-I and agroclavine) or prepared (setoclavine and lysergic acid) standards [20,21]. Setoclavine and lysergic acid were monitored by fluorescence with 310 nm excitation and 410 nm emission and quantified by comparing peak areas to an external standard curve of ergonovine maleate (Fisher Scientific, Pittsburgh, PA, USA), which has the same fluorophore. The values of these analytes must thus be considered as ‘relative to ergonovine’ as opposed to absolute. Chanoclavine-I and agroclavine were monitored by fluorescence at 272 nm/372 nm and quantified relative to an external standard curve of agroclavine (Fisher Scientific). The values for chanoclavine-I must thus be considered as relative to agroclavine as opposed to absolute. Differences in means between treatments were assessed with Student’s *t* test, assuming unequal variance and an alpha of 0.05. Statistical analyses were conducted in JMP version 17 (SAS, Cary, NC, USA).

### 2.2. Localization of easT in A. fumigatus

The promoter from the *A. fumigatus* glyceraldehyde-phosphate dehygrogenase gene (*gpdA*) was used to drive constitutive expression of fluorescent reporter fusion products. This promoter (nucleotides 3,418,743 to 3,419,708 of GenBank accession number VBRB01000006.1) was amplified from *A. fumigatus* genomic DNA with primer combination 4 (Table S1) to generate the *A*. *fumigatus gpdA* promoter fragment with restriction sites for *Sac*I and *Spe*I near the termini of the amplicon. Plasmid pBChygro (Fungal Genetics Stock Center, Manhattan, KS, USA) [23] was used as a template with primer combination 5 to amplify the hygromycin resistance gene along with its accompanying promoter and 3′-UTR. The phleomycin resistance gene and flanking regions were similarly amplified from pBCphleo plasmid (Fungal Genetics Stock Center) [23] using primer combination 6. Primer pairs 5 and 6 included restrictions sites for *Sbf*I near both termini, allowing for digestion, purification, and subsequent ligation into downstream vectors.

pDS221 plasmid (Addgene plasmid 34981) [24] was used with primer combination 7 (Table S1) to amplify the mCherry gene and to introduce an additional set of nucleotides (TCGAAGTTGTAG) coding for the peroxisomal targeting signal and a stop codon. Additionally, the primers included restriction sites for *Spe*I and *Sal*I. The plasmid pAG426GPD-EYFP-*ccdB* (Addgene plasmid 14348) was used as a backbone for mCherry expression experiments. The native promoter in this plasmid was excised via double-digestion using *Sac*I/*Spe*I. The *A. fumigatus gpdA* promoter was similarly double-digested and purified before both fragments were ligated together, generating AfGPDH-EYFP-*ccdB*. One Shot^™^ *ccdB* Survival^™^ 2 T1^R^ cells (Thermo Scientific, Waltham, MA, USA) were used for propagation of this and all other plasmids containing the *ccdB* gene. Bacterial transformants were plated on LB medium (per liter, 10 g tryptone, 5 g yeast extract, 5 g NaCl, and 15 g agar) supplemented with chloramphenicol (25 μg/mL). These and all other plasmid products were harvested from selected colonies and purified by plasmid miniprep. AfGPDH-EYFP-*ccdB* and the hygromycin cassette fragment were then digested using *Sbf*I, purified, and ligated together, generating AfGPDH-EYFP-*ccdB*-HygR. The EYFP-*ccdB* portion of this plasmid was then excised using *Spe*I/*Sal*I, while the same enzyme pair was used to treat the mCherrySKL fragment. The gel-purified fragments were then ligated together, generating AfGPDH-mCherrySKL-HygR. This plasmid did not require *ccdB*-competent *E. coli* cells, and transformants were plated on LB medium supplemented with ampicillin (100 μg/mL).

*Aspergillus leporis* isolate NRRL 3216 DNA was used as a template with primer combination 8 (Table S1) to amplify *A. leporis easT* without the native stop codon (nucleotides 24,443 to 26,176 from Genbank accession number SWBU01000104.1) [6]. Primer combination 9 was used to amplify the same region, albeit with the native stop codon and 3′-UTR (nucleotides 24,187 to 26,176 from the same accession). The plasmids pAG426GPD-*ccdB*-Cerulean and pAG426GPD-Cerulean-*ccdB* (Addgene plasmids 14396 and 14420, respectively) were digested using *Sac*I/*Spe*I and gel-purified. Both fragments were ligated with the *A. fumigatus gpdA* promoter fragment, generating AfGPDH-*ccdB*-Cerulean and AfGPDH-Cerulean-*ccdB*. The phleomycin resistance cassette fragment was then digested by *Sbf*I before being ligated into both plasmids, generating AfGPDH-*ccdB*-Cerulean-PhleoR and AfGPDH-Cerulean-*ccdB*-PhleoR. These plasmids served as recipients in exponential megapriming PCR reactions [25,26] using primer sets 10 and 11 together with the products generated from combinations 8 and 9, respectively, which served as the megaprimers. The linear products were then purified, phosphorylated, and self-ligated, generating AfGPDH-*easT*-Cerulean-PhleoR and AfGPDH-Cerulean-*easT*-PhleoR plasmids for *E. coli* transformation. Primer combinations 12 and 13 were used to verify correct assembly of the AfGPDH-*easT*-Cerulean and AfGPDH-Cerulean-*easT* portions, respectively, and their presence in fungal transformants.

To observe the expression of fluorescent proteins, 5000 conidia of each mutant strain were suspended in 200 μL of malt extract medium broth in wells of Nunc^™^ Lab-Tek^™^ II chamber slides (Thermo Scientific) and incubated in humidified chambers in total darkness at 37 °C for 24 h. Each chamber was rinsed twice with 200 μL PBS, mounted using SlowFade Glass Soft-set Antifade medium (Thermo Scientific) and a #1.5 coverslip, and then the edges of the coverslip were sealed with clear nail polish. A Zeiss LSM 710 Confocal microscope with AiryScan equipped with an AxioObserver.Z1 (Carl Zeiss AG, Oberkochen, Germany) was used to image slides of mounted cells. ZEN lite 2011 software was used for image generation and analysis (Carl Zeiss AG).

### 2.3. Knockout of easT in A. leporis

We knocked out *easT* in *A. leporis* isolate NRRL 3216 by application of the transient CRISPR-Cas9-based approach described by Jones and Panaccione [18]. The single guide RNA (sgRNA) was synthesized from the template 5′-TTCTAATACGACTCACTATAGACTGTGGCGGGACCGCTGGTGTTTTAGAGCTAGA-3′ (where the target sequence is underlined) with the EnGen sgRNA synthesis kit (New England Biolabs, Ipswich, MA, USA) and purified with the Monarch RNA clean-up kit (New England BioLabs). The sgRNA was complexed with EnGen Spy Cas9 NLS (New England BioLabs) and co-transformed with pBCphleo into *A. leporis* protoplasts prepared as previously described [18]. Transformants were purified to nuclear homogeneity by culturing from single conidia and screened for mutation in *easT* by PCR with primer combination 14 (Table S1). PCR products differing in size from that obtained from wild-type *A. leporis*, as assessed through agarose gel electrophoresis, were purified and sequenced by Sanger technology at Eurofins Genomics (Louisville, KY, USA).

Ergot alkaloid profiles of an *easT* knockout mutant and wild-type *A. leporis* were analyzed by growing cultures (*n* = 12 per strain) in 500 μL of sucrose-yeast extract medium (per liter: 20 g sucrose, 10 g yeast extract, and 1 g MgSO_4_-heptahydrate) in 2 mL screw cap microcentrifuge tubes for 8 days at 30 °C. The liquid phase was removed by pipetting from below the surface mat of mycelium and conidia; its volume was measured, and an equal volume of methanol was added to prepare it for HPLC analysis. The solid phase was transferred to a pre-weighed microcentrifuge tube, dried in a speed vac, weighed, and extracted with 1 mL of methanol. Ergot alkaloids were identified and analyzed by HPLC with fluorescence detection, as described above in Section 2.1 and in previous publications [6,18]. Lysergic acid and lysergic acid amides (lysergyl-alanine, ergonovine, LAH, and ergine) were monitored by fluorescence at 310 nm/410 nm and measured by comparing peak areas to a standard curve prepared from ergonovine maleate (Fisher Scientific); thus (with the exception of ergonovine), the concentration values for these alkaloids must be considered as relative to ergonovine as opposed to absolute. Chanoclavine-I was monitored by fluorescence at 272 nm/372 nm and quantified relative to an external standard curve of chanoclavine-I (Alfarma; Prague, Czech Republic). Statistical comparisons were made with Student’s *t* test, as described above (Section 2.1). Culturing and HPLC analysis were repeated with similar results.

## 3. Results and Discussion

### 3.1. Analysis of easT by Heterologous Expression in A. fumigatus

At the initiation of this project, we did not have a genetic transformation system for *A. leporis*; thus, we began functional analyses of the product of *easT* by heterologous expression. The expression host chosen was a strain of *A. fumigatus* (referred to here as *A. fumigatus* strain LA) that had been previously modified to produce lysergic acid as its ergot alkaloid pathway end-product, while accumulating the intermediates and shunt products shown prior to lysergic acid in Figure 1 [21]. This strain was selected as a potential target for improvement by expression of the hypothesized ergot alkaloid transporter encoded by *easT* because it produced a pharmaceutically relevant ergot alkaloid (lysergic acid) but yields were low [8,21] and the majority of the lysergic acid produced was retained in the solid phase of the fungus, as opposed to being secreted into the growth medium [8].

**Figure 2.**
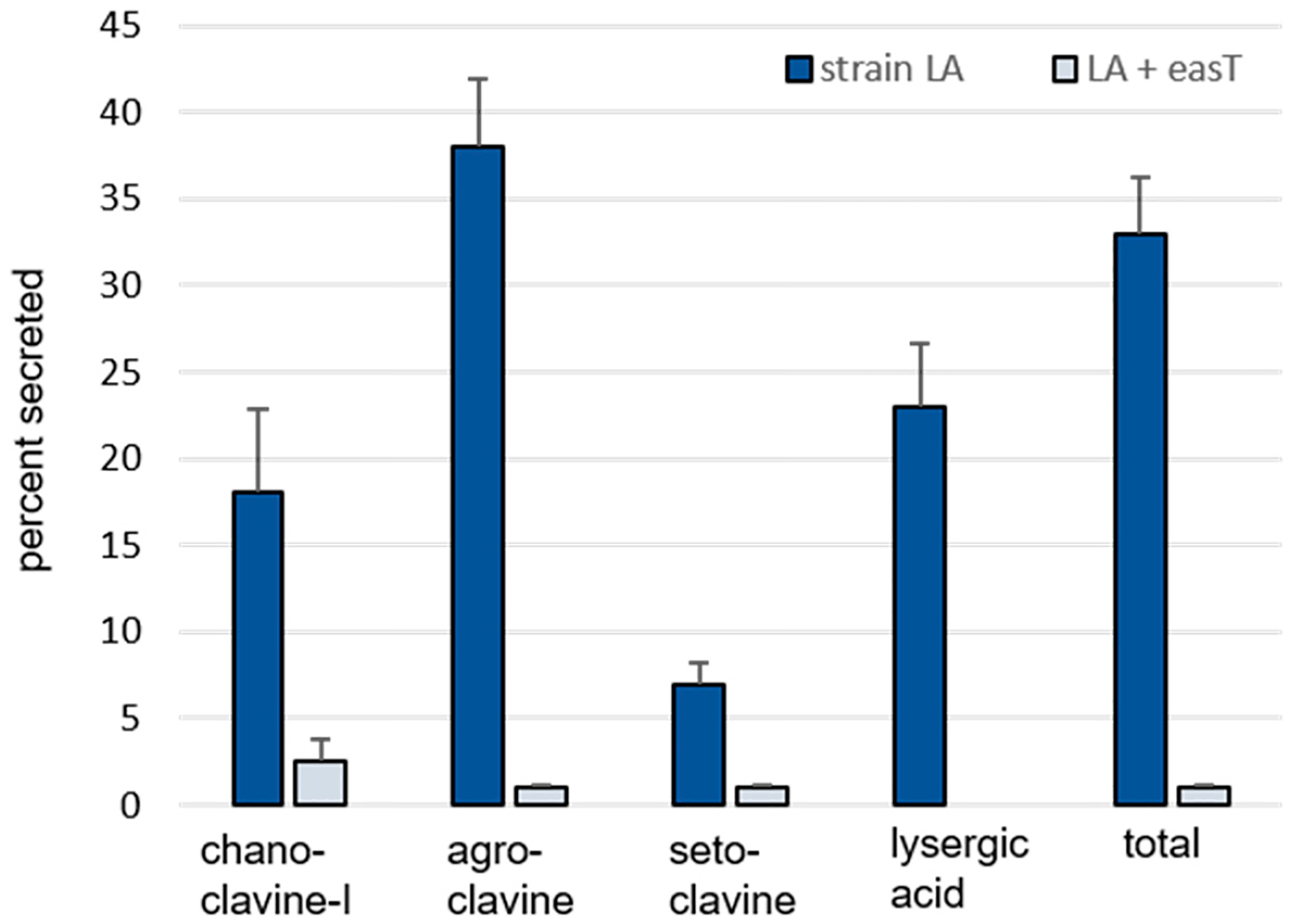
Percent of each measured ergot alkaloid secreted from *A. fumigatus* strain LA compared to an *easT*-expressing transformant of strain LA (LA + *easT*). Bars represent means of six samples per treatment, and error bars represent standard error. The percent of each ergot alkaloid secreted was greater in the wild type than in the *easT*-expressing strain, according to Student’s *t* tests (*p* < 0.05).

A derivative of *A. fumigatus* strain LA transformed with an *easT*-expression construct (Figure S1) accumulated significantly more ergot alkaloids than its parent strain but secreted a lower proportion of those alkaloids (Table 1, Figure 2). The transformant expressing *easT* accumulated significantly more chanoclavine-I (*p* = 0.0009), agroclavine (*p* = 0.0008), and setoclavine (an oxidation product of agroclavine) (*p* = 0.0015) in the solid phase of the culture than did *A. fumigatus* strain LA, with agroclavine, the precursor to lysergic acid, accumulating to 16-fold higher concentrations in the *easT*-expressing strain (Table 1). Surprisingly, no lysergic acid was detected in the *easT*-expressing strain. The liquid phases of the *easT*-expressing strain had lower concentrations of ergot alkaloids than did cultures of the parent strain, but the magnitude of the decrease in the liquid phase (4.5-fold) was smaller than the magnitude of the increase observed in the solid phase of the *easT*-expressing strain (12.7-fold) (Table 1). The overall mean yield of ergot alkaloids for combined phases of *easT*-expressing cultures was significantly higher (*p* = 0.0008), with 5410 nmol/g of fungus in the *easT*-expressing strain compared to 616 nmol/g in the parent strain. As indicated by comparison of solid and liquid phases listed in Table 1, the *easT*-expressing strain retained a higher proportion of each of its ergot alkaloids than did the parent strain (*p* < 0.05) (Figure 2).

One interpretation of these data is that the product of *easT* facilitated the conversion of early pathway intermediates into chanoclavine-I and agroclavine but, at least in this engineered system, inhibited the conversion of agroclavine to lysergic acid. A possible mechanism for this inhibition might be through compartmentalizing substrates and/or enzymes in different subcellular locations. In previous studies in which ergot alkaloid biosynthetic genes from the lysergic acid or dihydrolysergic acid branches of the ergot alkaloid pathway were expressed in *A. fumigatus*, investigators noted low turnover of intermediate substrates by the products of the introduced genes [8,15,21]. The introduction of EasT into the system appears to have allowed enzymes from early pathway steps up through those required for agroclavine synthesis better access to their substrates but contained agroclavine in such a way that it was not accessible to the clavine oxidase encoded by *cloA*. This final step in lysergic acid synthesis was previously engineered into *A. fumigatus* strain LA [21] and may have yielded the modest amount observed in that parent strain due to a lack of compartmentalization of agroclavine in the absence of EasT. A second important observation from this experiment is that the data do not support a role for the product of *easT* in the secretion of ergot alkaloids from the producing fungus into the growth medium. In fact, the *easT*-expressing strain appeared to have sequestered alkaloids such that they were not secreted to the same degree as they were in the parent strain.

### 3.2. Localization of the Product of easT Heterologously Expressed in A. fumigatus

**Figure 3.**
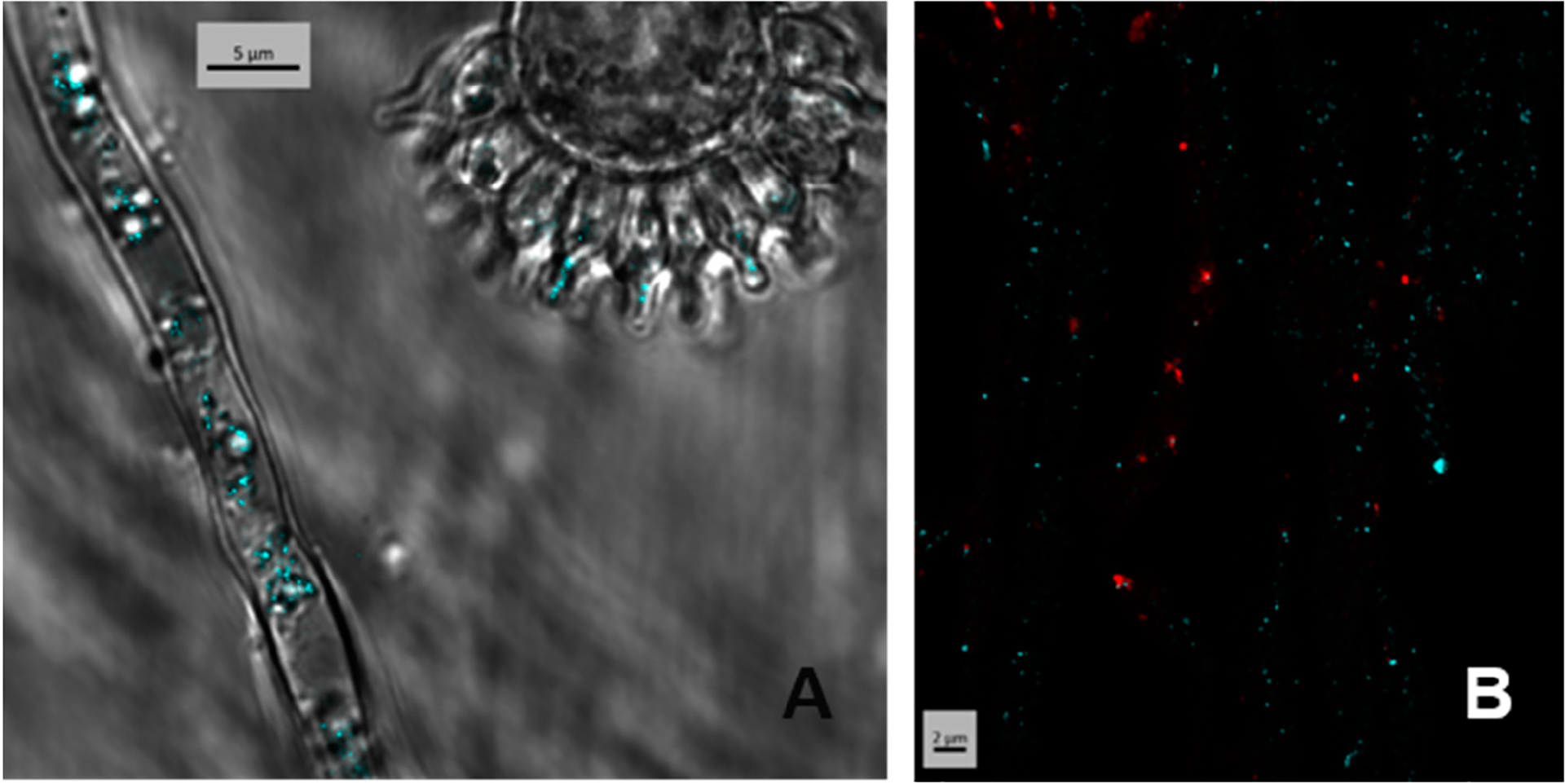
N-terminally labeled CFP-EasT observed within the conidiophore stalk, phialides, and conidia of A. fumigatus (**A**). N-terminally labeled CFP-EasT (blue) localizing separately from peroxisomally localized mCherry (red) in hyphae of *A. fumigatus* (**B**).

To investigate the localization of the product of *easT* in transformed *A. fumigatus*, we expressed CFP-labeled versions of the protein. EasT labeled with CFP at either the amino or carboxy terminus was observed in small vesicle-like structures internal to the mycelium, conidiophores, and conidia of the fungus (Figures 3A and S2A). The intracellular components containing EasT labeled at the amino terminus or carboxy terminus were distinct from peroxisomes, which were independently labeled with mCherry (Figures 3B and S2B). No labeling of the plasma membrane was observed, consistent with the lack of evidence for EasT contributing to the secretion of ergot alkaloids when expressed in A. fumigatus. Contiguity of the CFP marker with EasT was confirmed by Western blot analysis, in which CFP antibody reacted with a protein of the size expected for CFP fused to EasT (82 kDa) (Figure S3). The apparent association of EasT with the endomembrane system is consistent with its strong amino acid sequence identity with members of the major facilitator superfamily [6] and the presence of 12 membrane-spanning domains predicted in a Kyte and Doolittle hydropathy plot (Figure S4). Twelve membrane-spanning domains represent the most frequently observed topology for this family of transporter [27].

### 3.3. Analysis of easT by Gene Knockout in A. leporis

Transient introduction of Cas9 with an *easT*-targeted sgRNA resulted in a knockout of *easT* in *A. leporis*, as evidenced by PCR and Sanger sequence analysis of a resulting transformant (Figure S5). The modified locus was cut three-bp 5′ of the intended PAM site, and fragments of pBCphleo, which had been introduced by co-transformation as a selectable marker, were ligated into the cut site during repair. Knockout of *easT* in *A. leporis* reduced internal (solid phase) concentrations of the four amide derivatives of lysergic acid: lysergyl-alanine, ergonovine, LAH, and ergine (Table 2). The magnitudes of the reductions were moderate (from 23% to 50% for individual alkaloids) but statistically significant in each case (*p* < 0.05). Concentrations of earlier pathway intermediates chanoclavine-I and lysergic acid did not differ significantly in the *easT* knockout compared to the wild type. Agroclavine, the immediate precursor to lysergic acid, was not detected in wild type or knockout, indicating efficient turnover of agroclavine to lysergic acid (with or without EasT). Other intermediates not listed in Table 2 were also not detected, indicating efficient conversion of each of these intermediates to the next step of the pathway. Concentrations of ergot alkaloids in the liquid phases did not differ significantly between wild-type *A. leporis* and its *easT* knockout, with the exception of a higher concentration of ergine in the liquid phase of the knockout (*p* = 0.03). Interpretation of the amount of ergine in the liquid phase is confounded by the fact that ergine is derived from hydrolysis of LAH (Figure 1), and such hydrolysis may occur prior to and after secretion of LAH. In an attempt to account for these confounding factors, values for LAH and ergine were combined. The total of LAH + ergine did not differ significantly in the liquid phases of wild-type and knockout strains (*p* = 0.14) (Table 2), consistent with the lack of difference observed with other ergot alkaloids in culture fluids. In the solid phase, however, the total of LAH + ergine was lower in the *easT* knockout compared to wild type (*p* = 0.04), consistent with the reduction in concentration of other lysergic acid amides noted in the solid phase of cultures of the *easT*knockout. When parsed this way, the data indicate that hydrolysis of LAH to ergine may be lower in the liquid phase of the *easT* knockout.

**Figure 4.**
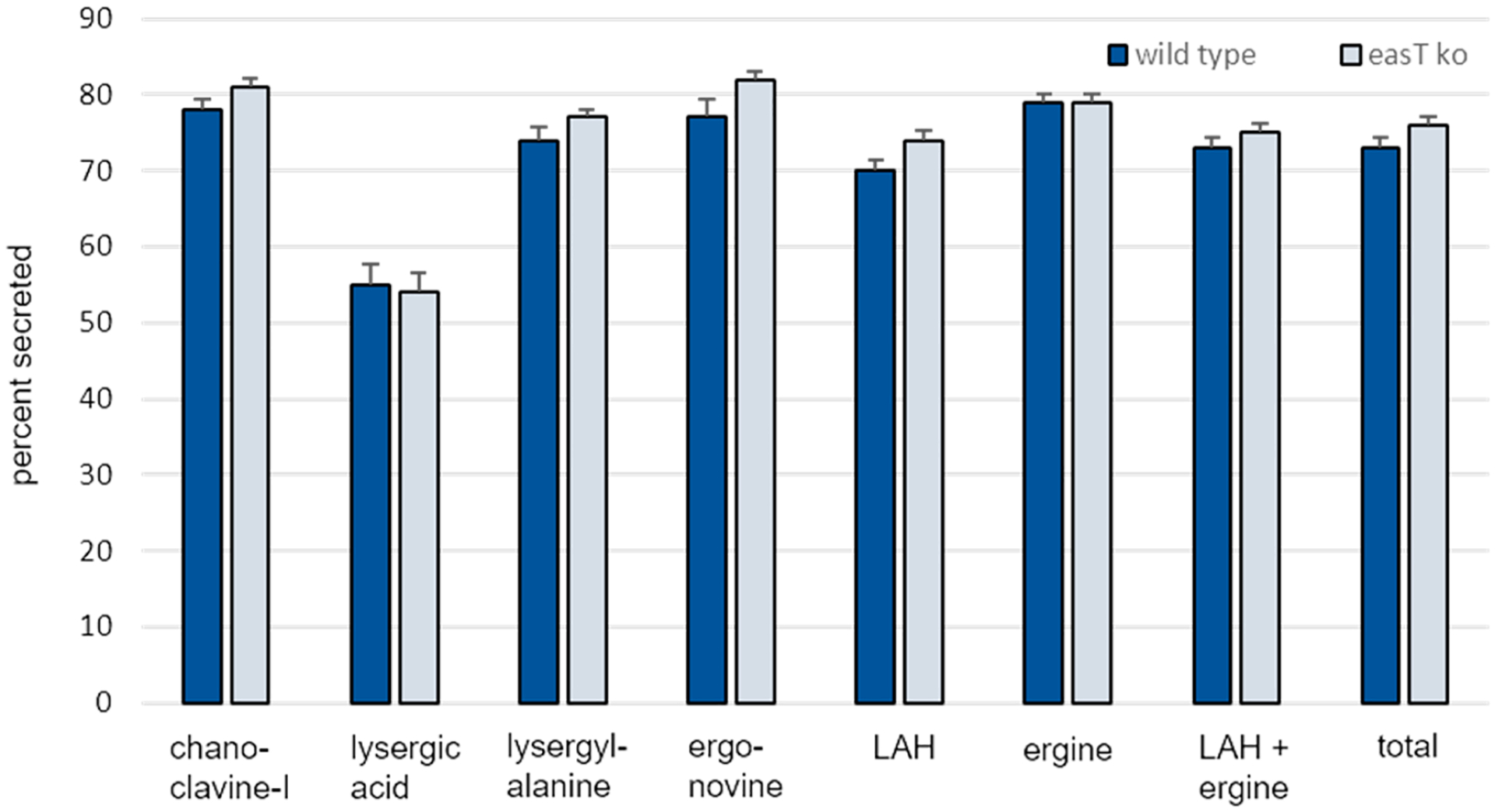
Percent of each measured ergot alkaloid secreted from *A. leporis* and an *easT* knockout mutant. Bars represent means of 12 samples per treatment, and error bars represent standard error. The percent of each ergot alkaloid secreted differed significantly (*p* < 0.05, in a Student’s *t* test) only for LAH. Refer to text for discussion of this point.

Overall secretion of ergot alkaloids into the growth medium did not differ significantly in the *easT* knockout strain (76%) compared to the wild type (73%) (*p* = 0.16) (Figure 4). Secretion of individual ergot alkaloids also did not differ significantly (*p* > 0.05), except for LAH, which appeared to be secreted at a higher rate in the *easT* knockout (74%) than in wild type (70%) (*p* = 0.03). The minor magnitude of this difference in secretion (4%) and the previously mentioned issue of hydrolysis (or lack thereof) of LAH to ergine are relevant to consider with respect to this point. When considered together with its hydrolysis product ergine, values for secretion of LAH + ergine did not differ significantly between strains (*p* = 0.16).

Altogether, the data do not support a role for a properly functioning EasT in the secretion of ergot alkaloids. The greater impact of EasT on the internal concentrations of ergot alkaloids in *A. leporis* as compared to concentrations of external ergot alkaloids suggests that the secretion of ergot alkaloids occurs rapidly and was not deterred by the slightly lower concentrations of lysergic acid derivatives detected in the *easT* knockout strain.

### 3.4. Differences in Ergot Alkaloid Accumulation in Engineered A. fumigatus Compared to A. leporis

Differences were noted in the results of experiments that added *easT* to an engineered strain of *A. fumigatus* or knocked out *easT* in its source fungus, *A. leporis*. One important difference in these fungi relevant to these results is that *A. leporis*, the source of *easT*, naturally produces lysergic acid amides and secretes them into the growth medium [6], whereas *A. fumigatus* naturally produces a different branch of ergot alkaloids (fumigaclavines) and retains those alkaloids in the solid phase [7]. This difference in the ultimate location of the pathway products may indicate a difference in subcellular location of pathway intermediates and enzymes in the two fungi. A second important point to consider with respect to the function of EasT in these experiments is that in experiments with *A. fumigatus* strain LA, *easT* was introduced into a system that had been previously modified to produce agroclavine and lysergic acid (instead of its natural fumigaclavines) by heterologously expressing genes originating from an *Epichloë* species [21]. Issues with localization of enzyme and substrate had been suspected in this system based on poor turnover of substrates to products [21]. These differences may be at least partly responsible for EasT appearing to interact with intermediates differently in the two systems.

The fate of agroclavine in the two different fungi should be considered in light of the two different experimental approaches. Knockout of *easT* in *A. leporis* did not negatively affect turnover of agroclavine to lysergic acid, indicating that EasT does not transport agroclavine in the wild type and/or that agroclavine was already localized favorably relative to CloA (the enzyme that oxidizes agroclavine to lysergic acid) in *A. leporis*. When *easT* of *A. leporis* was expressed in *A. fumigatus* strain LA, it did not help convert agroclavine to lysergic acid, indicating a failure to recognize agroclavine as substrate or to bring together agroclavine with the heterologously expressed CloA from *Epichloë* sp. Both results are consistent with a lack of association of EasT with agroclavine. In *A. leporis*, EasT may not be required to bring agroclavine to the same location as CloA; however, in the engineered strain of *A. fumigatus*, agroclavine and CloA may have been in different locations, and EasT was unable to bring them together. Some of the agroclavine that accumulated in the engineered strain of *A. fumigatus* was oxidized to setoclavine, presumably by endogenous peroxidases, as has been described previously [19–21]. Setoclavine was not detected in wild-type or *easT* knockout strains of *A. leporis*, presumably because agroclavine was efficiently converted to lysergic acid in these strains.

## 4. Conclusions

Evidence from three lines of enquiry, including (1) heterologous expression of *easT* in an engineered system (previously modified, lysergic acid-producing *A. fumigatus*); (2) subcellular localization of fluorescently labeled EasT; and (3) knockout of *easT* in its native fungus, *A. leporis*, is consistent with a role for EasT as an intracellular transporter of ergot alkaloid pathway intermediates and products. The data support previous studies indicating differential subcellular location of steps in the ergot alkaloid pathway [9,10] and indicate that the product of *easT* facilitates but is not absolutely required for ergot alkaloid synthesis.

## Supplementary Material

Jones et al 2024 supplement

## Figures and Tables

**Table 1.** Accumulation of ergot alkaloids in *Aspergillus fumigatus* strain LA and an *easT*-expressing transformant.

	nmol Alkaloid/g Dry Weight Fungus (Mean ± SE)			
	Solid Phase		Liquid Phase	
Alkaloid	*A. fumigatus* Strain LA	Strain LA + *easT*	*A. fumigatus* Strain LA	Strain LA + *easT*
Chanoclavine-I	66 ± 9 A^1^	174 ± 18 B	14 ± 1 a	4 ± 0.4 b
Agroclavine	302 ± 51 A	4891 ± 634 B	171 ± 11 a	37 ± 8 b
Setoclavine	37 ± 4 A	303 ± 43 B	3 ± 0.3 a	2 ± 0.2 b
Lysergic acid	19 ± 4	n.d.^2^	5 ± 0.5	n.d.
Total	424 ± 67 A	5367 ± 690 B	192 ± 12 a	43 ± 9 b

1 Means for individual alkaloids that differ significantly in a Student’s *t* test (*p* < 0.05) are indicated with a different letter. Solid and liquid phases were compared separately and thus are labeled with upper case versus lower case letters. Mean comparisons are separate for each alkaloid (row) and each culture phase (paired columns).

2 Not detected.

**Table 2.** Accumulation of ergot alkaloids in *Aspergillus leporis* and an *easT* knockout transformant.

	nmol Alkaloid/g Dry Weight Fungus (Mean ± SE)			
	Solid Phase		Liquid Phase	
Alkaloid	Wild Type	*easT* Knockout	Wild Type	*easT* Knockout
Chanoclavine-I	6 ± 0.7 A^1^	4 ± 0.4 A	20 ± 2 a	19 ± 0.7 a
Lysergic acid	2 ± 0.2 A	2 ± 0.2 A	3 ± 0.3 a	2 ± 0.2 a
Lysergyl-alanine	4 ± 0.3 A	3 ± 0.2 B	11 ± 0.9 a	9 ± 0.4 a
Ergonovine	2 ± 0.1 A	1 ± 0.1 B	6 ± 0.7 a	6 ± 0.3 a
LAH	185 ± 17 A	144 ± 35 B	427 ± 40 a	401 ± 21 a
Ergine	61 ± 6 A	46 ± 4 B	237 ± 20 a	174 ± 6 b
LAH + ergine	246 ± 22 A	189 ± 13 B	665 ± 55 a	575 ± 23 a
Total	259 ± 23 A	200 ± 14 B	705 ± 59 a	611 ± 25 a

1 Means for individual alkaloids that differ significantly in a Student’s *t* test (*p* < 0.05) are indicated with a different letter. Solid and liquid phases were compared separately and thus are labeled with upper case versus lower case letters. Mean comparisons are separate for each alkaloid (row) and each culture phase (paired columns).

## Data Availability

All data are presented in the article and Supplementary Materials.
