# Supplementary material for: A Major Facilitator Superfamily Transporter Contributes to Ergot Alkaloid Accumulation but Not Secretion in Aspergillus leporis
Source: Appl Microbiol (Basel). Author manuscript; Available in PMC 2024 Jul 25. (PMC11271707; doi:10.3390/applmicrobiol4010028)
Supplement: Jones et al 2024 supplement [file NIHMS2007706-supplement-Jones_et_al_2024_supplement.docx]

**Supplementary Materials for**

**A major facilitator superfamily transporter contributes to ergot alkaloid accumulation but not secretion in *Aspergillus leporis***

Abigail M. Jones, Kyle A. Davis, and Daniel G. Panaccione #

Division of Plant and Soil Sciences, West Virginia University,

Morgantown, WV 26506 USA

# corresponding author: Daniel G. Panaccione

**Email**: danpan@wvu.edu

**This supplemental file includes:**

Table S1: Primers and PCR Protocol Information

Figure S1: Detection of the *easT*-expression construct in transformants of *A. fumigatus* strain LA

Figure S2: Localization of EasT-CFP and mCherry-SKL fusion proteins in transformants of
 *A. fumigatus* strain LA

Figure S3: Confirmation of mRNA and protein expression

Figure S4: Kyte-Doolittle hydropathy plot derived from EasT amino acid sequence

Figure S5: PCR and DNA sequence analyses of *easT* knockout in *A. leporis*

**Table S1. Primers and PCR Protocol Information**

| Primer pair | Primer sequences (5′ to 3′)*^a^* | Product (length) | Annealing temperature (°C), Extension time (s) |
| --- | --- | --- | --- |
| 1 | CATGCTTCTAATCCACCAAGTAC +  GACAGCCGAAATAACGTACCATGGTGCGGAGTGCCTAC | *A*. *fumigatus* *easA* promoter with 22-nt overlap with *A. leporis* *easT* (812 bp) | 63, 30 |
| 2 | GTAGGCACTCCGCACCATGGTACGTTATTTCGGCTGTCC + TAGGAACAATGCATCTCAAG | *A. leporis easT* with 16-nt overlap with *easA* promoter from *A. fumigatus* (2050 bp) | 60, 60 |
| 3 | CATGCTTCTAATCCACCAAGTAC + TAGGAACAATGCATCTCAAG | *A*. *fumigatus* *easA* promoter fused to *A. leporis* *easT* (2824 bp) | 60, 90 |
| 4 | AGTCGGAGCTCCGCAGATTCTAGAAGTCCTG +  GCTAGACTAGTTGTGTAGATTCGTCTGGTAC | *A*. *fumigatus* *gpdA* promoter (990 bp) | 61, 30 |
| 5 | GTCACCTGCAGGTCCGTCTCCATTGGCTCTTG +  AGCTCCTGCAGGCTATTCCTTTGCCCTCGGAC | Hygromycin resistance gene including promoter and 3’UTR (1851 bp) | 64, 60 |
| 6 | GTCACCTGCAGGGTACCCGGGGATCTTTCGAC +  AGCTCCTGCAGGTACATGCGTACACGCGTCTG | Phleomycin resistance gene including promoter and 3’UTR (2945 bp) | 65, 90 |
| 7 | GCTAGACTAGTATGGTGAGCAAGGGCGAG +  GGTCAGTCGACCTACAACTTCGACTTGTACAGCTCGTCCATGC | mCherry gene with terminal nucleotides for -SKL amino acid sequence (742 bp) | 53, 60 |
| 8 | *ACAAGTTTGTACAAAAAAGCTGAACGAGAA*ATGGTACGTTATTTCGGCTG + *ACCACTTTGTACAAGAAAGCTGAACGAGAA*CCGTCAGCATGTCTTCGCTT | *A*. *leporis* *easT* without stop codon and 3’UTR and *attR1/2* overlaps (1793 bp) | 61, 60 |
| 9 | *ACAAGTTTGTACAAAAAAGCTGAACGAGAA*ATGGTACGTTATTTCGGCTG + *ACCACTTTGTACAAGAAAGCTGAACGAGAA*ATACAGATCCGGAGATGATG | *A*. *leporis* *easT* with stop codon and 3’UTR and *attR1/2* overlaps (2070 bp) | 60, 60 |
| 10 | GATGGGCTGCAGGAATTCGATATCAAGCTTAATGGTGAGC +  *ACCACTTTGTACAAGAAAGCTGAACGAGAA*CCGTCAGCATGTCTTCGCTT | Linear Af*easTCFP*-PhleoR plasmid from exponential megapriming PCR (12,348 bp) | 72, 390 |
| 11 | GCCGGTACCCAATTCGCCCTATAGTGAGTC + ACCACTTTGTACAAGAAAGCTGAACGAGAAATACAGATCCGGAGATGATG | Linear Af*CFPeasT*-PhleoR plasmid from exponential megapriming PCR (12385 bp) | 72, 240 |
| 12 | GTACGTTATTTCGGCTGTCC +  GTTACTTGTACAGCTCGTCC | A portion of *A. leporis easT*-CFP fusion (2512 bp genomic DNA, 2266 bp cDNA) | 61, 90 |
| 13 | CACATGAAGCAGCACGACTT +  CCCTTCTCTCTGGCTCGAG | A portion of CFP-*A. leporis easT* fusion for RT-PCR (1470 bp genomic DNA, 1224 bp) | 64, 60 |
| 14 | CAAGGTGCATCATCTGCCG +  CCCTTCTCTCTGGCTCGAG | *A. leporis* *easT* locus before (306 bp) and after (~2300 bp) knockout | 65, 90 |

*^a^* Underlines indicate unique restriction sites inserted to facilitate cloning of products: GAGCTC, *Sac*I; GTCGAC, *Sal*I; CCTGCAGG, *Sbf*I; ACTAGT, *Spe*I.


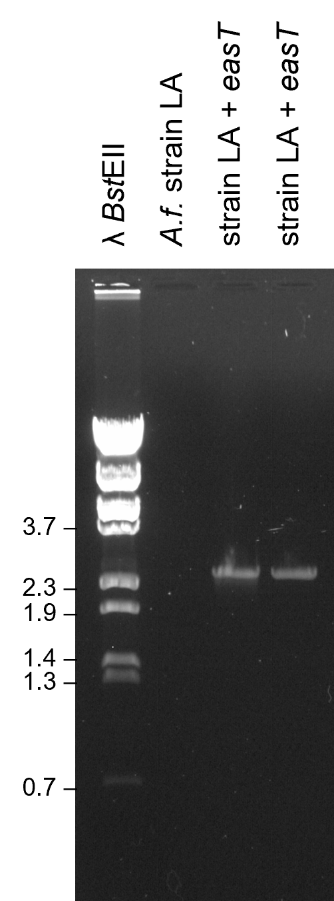


**Figure S1.** Detection of the *easT*-expression construct in transformants of *A. fumigatus* strain LA. PCR products (or lack thereof, in the case of the recipient strain) were obtained in reactions with primer combination 3 (Table S1). Sizes (in kb) of relevant fragments of *Bst*EII-digested bacteriophage lambda are indicated to the left of the gel.


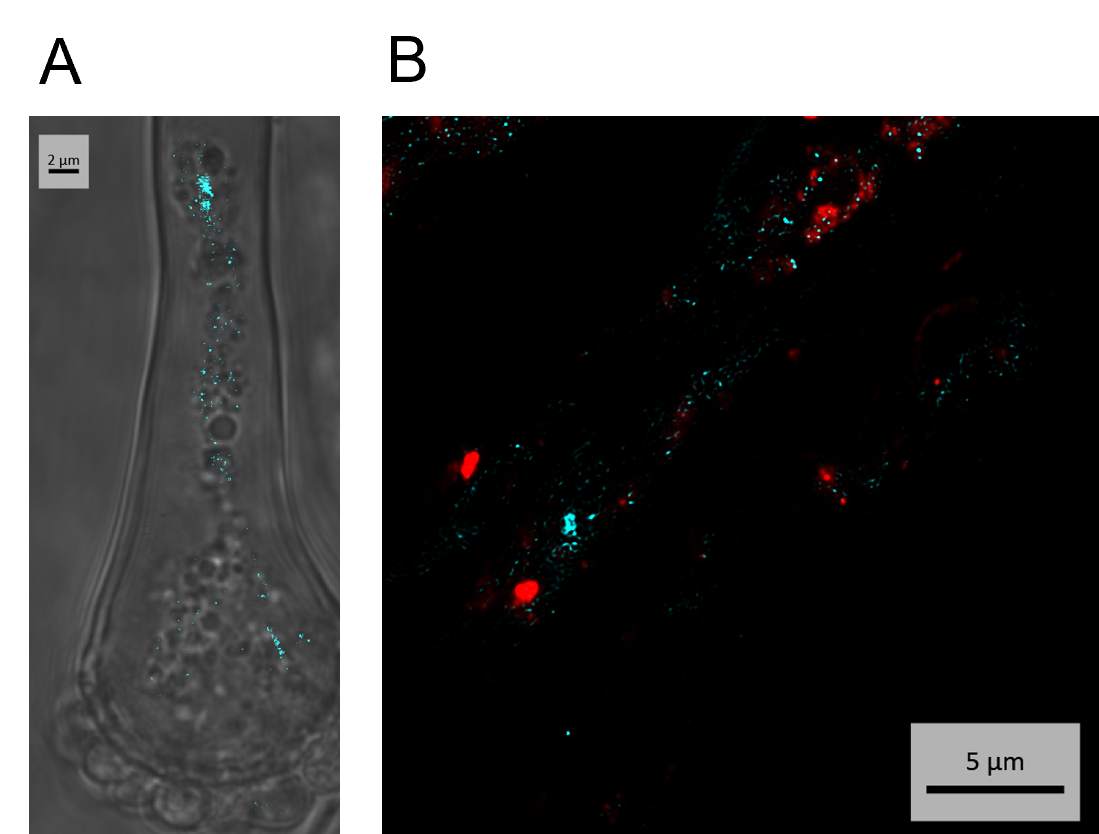


**Figure S2.** Localization of EasT-CFP and mCherry-SKL fusion proteins in transformants of *A. fumigatus* strain LA. (A) Localization of EasT-CFP and in a conidiophore visualized with overlaid fluorescence and differential interference contrast. (B) Localization of mCherry-SKL fusions (red) and EasT-CFP (blue) in hyphae of *A. fumigatus* strain LA.


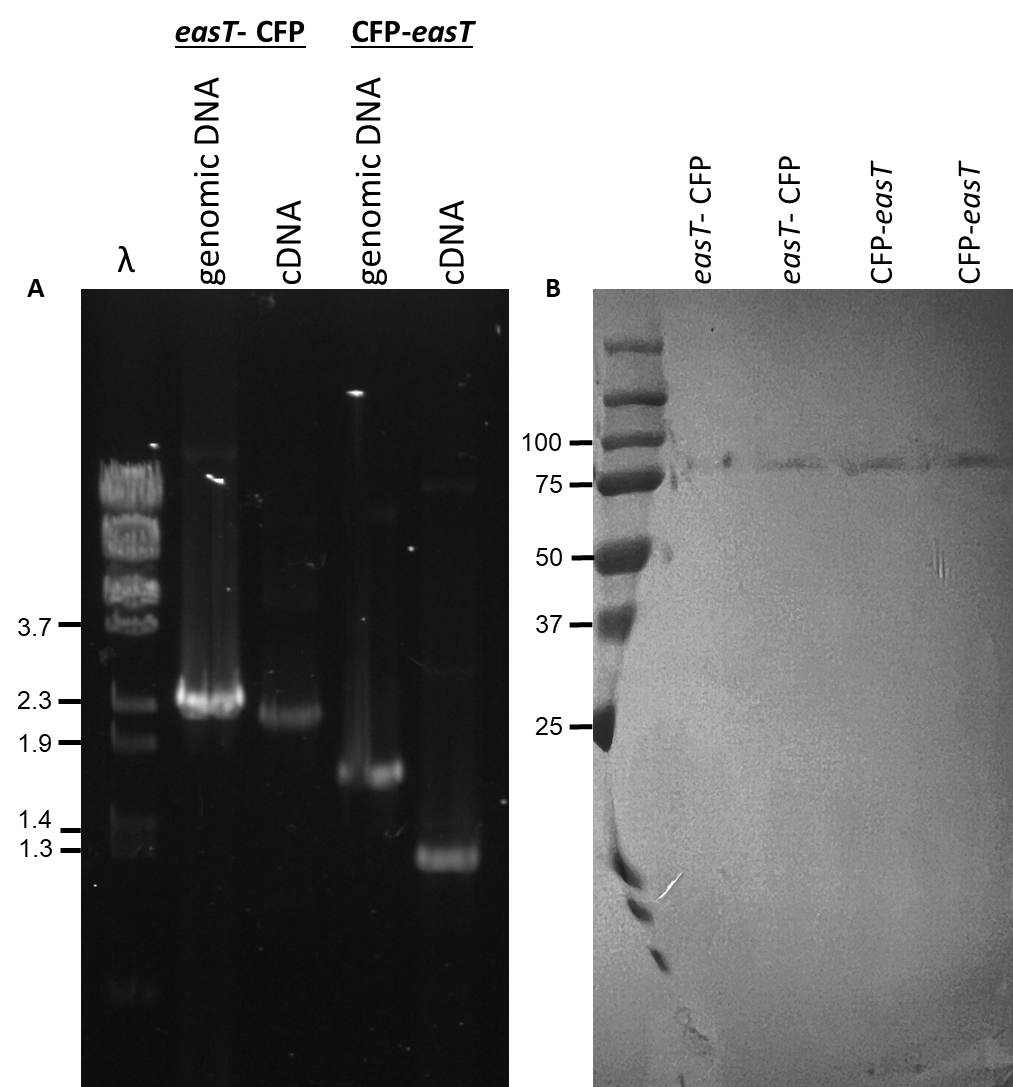


**Figure S3.** Confirmation of mRNA and protein expression. (A) DNA gel showing products amplified from genomic DNA and cDNA of *A. fumigatus* mutants expressing easT-CFP and CFP-easT fusions using primer combinations 12-13 (listed in Table S1), respectively. Sizes of relevant fragments from *Bst*EII-digested bacteriophage lambda DNA are indicated to the left. Gel was stained with ethidium bromide. (B) Western blot of duplicate membrane fractions from the same mutants as panel A. Sizes of Precision Plus pre-stained protein marker (Bio-Rad, Hercules, CA, USA) fragments in the first lane are indicated to the left of the blot. Size marker lane is distorted due to high amounts of detergent in membrane samples.


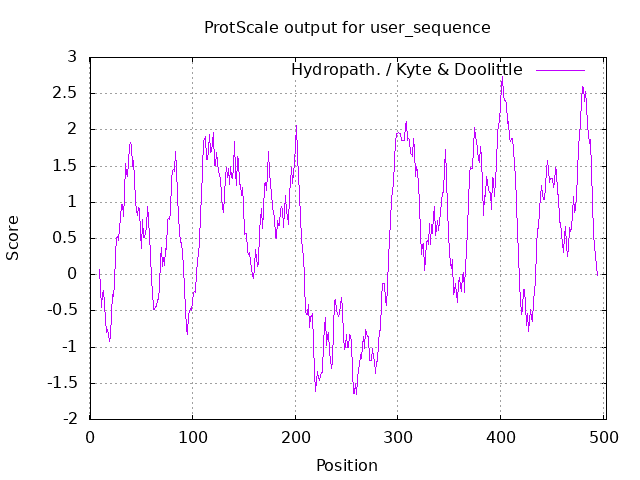

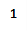

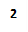

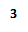

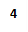

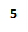

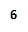

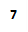

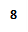

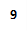

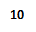

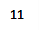

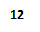


**Figure S4.** Kyte-Doolittle hydropathy plot derived from EasT amino acid sequence. A window size of 19 amino acids was used to search for transmembrane regions in this protein. The hydrophobic residues are shown above zero, whereas the hydrophilic residues are below zero. Derived hydrophobic regions are numbered. Plot was generated with ProtScale (web.expasy.org accessed 10 July 2023).


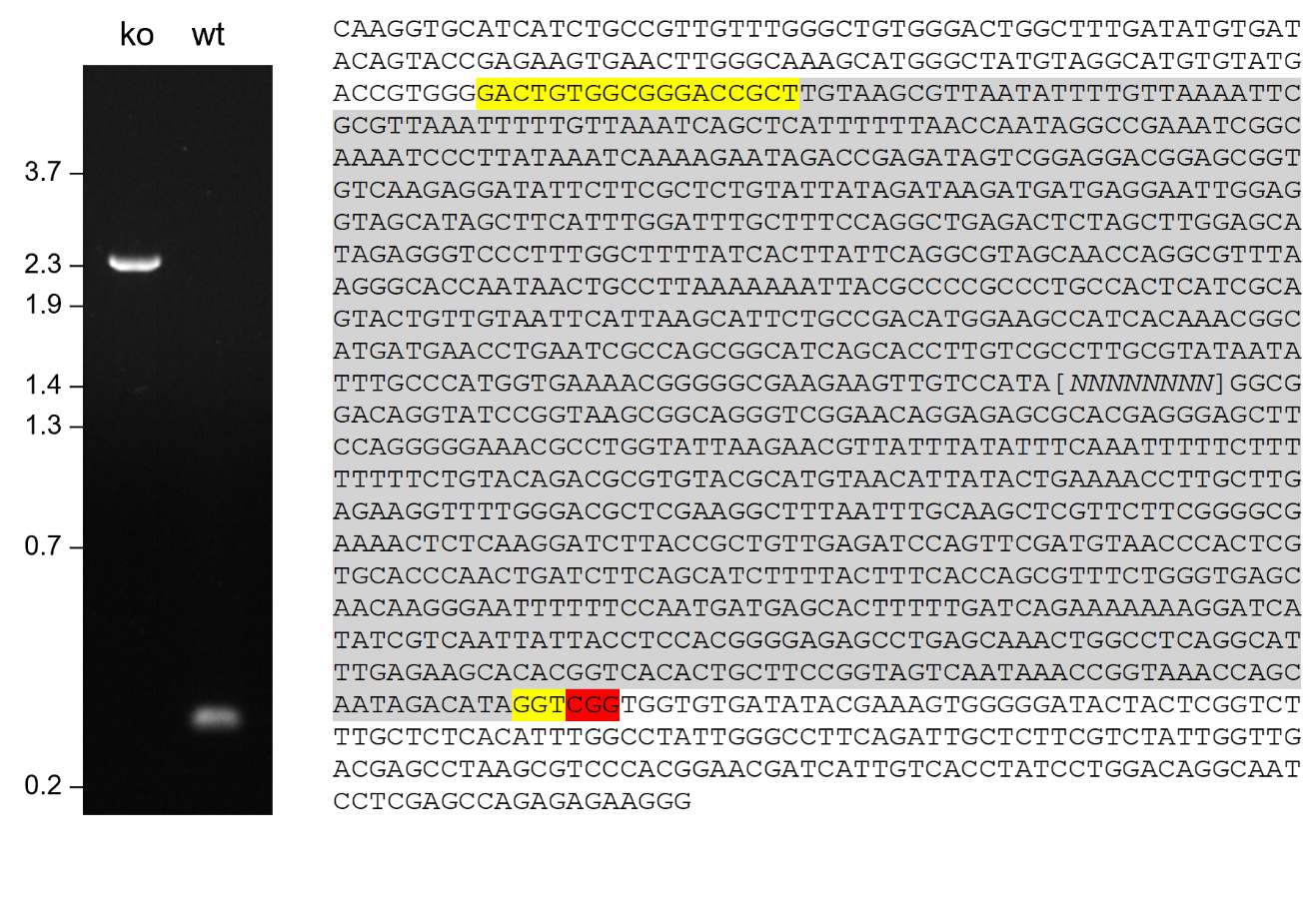


**Figure S5.** PCR and DNA sequence analyses of *easT* knockout in *A. leporis*. In the left panel, PCR products from a transformant (ko) and *A. leporis* strain NRRL 3216 (wt) were derived from reactions with primer combination 14 (Table S1). Relative mobility of relevant fragments (in kb) of *Bst*EII-digested bacteriophage λ are shown to the left of the gel. The right panel presents the DNA sequence of the *easT* locus after CRISPR-Cas9 mutagenesis. Unhighlighted sequence are part of *easT*. Sequences highlighted yellow are nucleotides from easT incorporated into the sgRNA, and the target sequence PAM site is highlighted red. Sequences of the pBCphleo selectable marker construct (incorporated into the locus during repair) are shaded gray. The abbreviation [*NNNNNNNN*] represents approximately 1000 nt of the insert that were omitted to simplify the presentation.
